# Pathological and molecular diagnosis of bilateral inguinal lymph nodes metastases from low-grade endometrial adenocarcinoma: a case report with review of the literature

**DOI:** 10.1186/s12885-017-3944-7

**Published:** 2018-01-02

**Authors:** Anna Myriam Perrone, Giulia Girolimetti, Simona Cima, Ivana Kurelac, Alessandra Livi, Giacomo Caprara, Donatella Santini, Paolo Castellucci, Alessio Giuseppe Morganti, Giuseppe Gasparre, Pierandrea De Iaco

**Affiliations:** 1 Oncologic Gynecology Unit, Sant’Orsola-Malpighi hospital, Bologna, Italy; 2 Department. Of Surgical and Medical Sciences (DIMEC), Medical Genetics Unit, S.Orsola-Malpighi Hospital, Bologna, Italy; 3 Radiotherapy Unit, Sant’Orsola-Malpighi hospital, Bologna, Italy; 4 Pathology Unit, Sant’Orsola-Malpighi hospital, Bologna, Italy; 5 Nuclear Medicine Unit, Sant’Orsola-Malpighi hospital, Bologna, Italy

**Keywords:** Endometrial carcinoma, Mitochondrial DNA sequencing, Inguinal lymph nodes metastasis

## Abstract

**Background:**

Extra-abdominal metastases in low grade endometrial carcinoma are rare events. Inguinal lymphatic spread occurs usually in advanced disease and is associated with abdominal lymph nodes involvement. To our knowledge, isolated inguinal lymph node metastases in patients with early endometrial carcinoma have never been described thus far.

**Case presentation:**

We present an uncommon case of inguinal lymph node metastasis in a 51-year old patient with early endometrial disease without other metastatic involvement. The metastatic loci were analyzed with the recently validated method of mitochondrial DNA sequencing to demonstrate clonality of the lesions.

**Conclusions:**

We describe the first case of inguinal metastasis from intramucous endometrial carcinoma; this case confirms the unpredictable spread of endometrial neoplasia and the importance of both patient’s history and physical examination in good clinical practice.

## Background

Endometrial Cancer (EC) is the sixth most common malignancy among females worldwide. Most patients usually present with low grade (72.4% grade 1 or 2) and with early stage (74.9% stage 1) diseases. Extra-uterine metastases are rare (8%), and pelvic and/or para-aortic lymph node involvement (LNI; stage III) occurs in 5 and 11% of cases respectively [1–3]. Inguinal lymph nodes metastases are considered as an unusual localization of distant metastases (stage IV) and generally occur in advanced uterine disease with positive pelvic nodes [4].

We describe an uncommon case of inguinal lymph node metastases in a patient with early endometrial disease without other metastatic localizations. The diagnosis of inguinal LNI from EC was performed by standard histological analysis on lymph node dissection specimens. Moreover, the recently validated method of mitochondrial DNA (mtDNA) sequencing [5] was used to confirmed the clonality of EC lesions.

## Case presentation

A 51-year-old woman was referred to our Gynecology Unit in July 2012, with hypermenorrhea and dysmenorrhea. The patient reported regular menstrual periods, two previous pregnancies with spontaneous deliveries and negative previous pap smear. Patient’s body mass index (BMI) was 23. The anamnesis of the patient was negative for any comorbidities. Family history of cancer was negative, too.

She reported a previous diagnostic hysteroscopy with negative endometrial sampling followed by 5-months progestins treatment without benefits.

On clinical examination and pelvic ultrasound her uterus appeared enlarged (length, anteroposterior and transverse diameter were 107x75x83 mm respectively) due to two intramural and sub-serosal uterine myomas (6 and 5 cm, respectively), with regular endometrium (thickness 8 mm) and ovaries.

Due to symptoms and age, hysterectomy was recommended. The patient declined surgical treatments and was switched to medical therapy with Gonadotropin Releasing Hormone (GnRH) analogue (Leuproreline 3.75 mg monthly intramuscular injections) for 6 months. Six months later, the patient reported recurrent symptoms and bilateral inguinal lymph node enlargement.

Further clinical examination and pelvic ultrasound showed unchanged uterine myomas, regular endometrium (5 mm) and enlarged, fixed right inguinal lymph node of 2 cm. Because of failure of medical therapy, surgical intervention was again proposed. In February 2013 the patient was referred to open surgical intervention: the exploration of all peritoneal surfaces and all abdominal cavities did not uncover any sign of malignancy or endometriosis. The uterus was enlarged (longitudinal diameter 10 cm), its surface was smooth and regular, ovaries were small and regular. Surface of peritoneum and all abdominal organs were free from disease and no enlarged pelvic and para-aortic lymph node were found. Surgical resection included hysterectomy, bilateral salpingo-oophorectomy, pelvic and bilateral inguinal lymphadenectomy and peritoneal washing for cytology. Intra-operative frozen sections of right inguinal lymph node dissection were positive for EC, although frozen sections of the endometrium were negative for EC. Due to cancer diagnosis, omentectomy and multiple peritoneal biopsies were performed. No post-operative complications were observed and the patient was discharged from the hospital in good conditions 6 days after surgery.

**Fig. 1:**
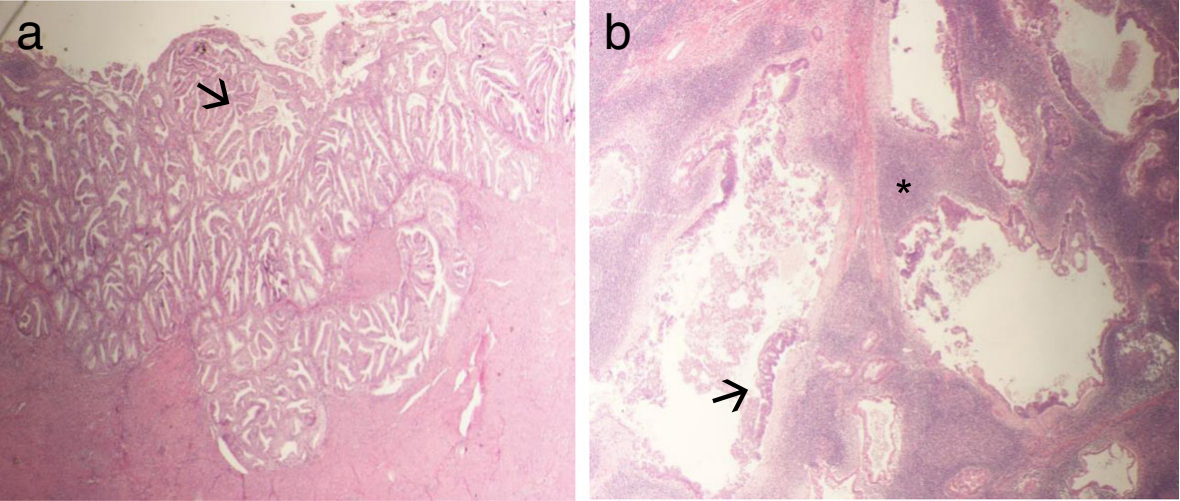
**A-** Endometrial endometrioid carcinoma. Low-grade endometrial carcinoma with well-preserved glandular architecture (arrow). **B-** Inguinal lymph node metastasis. Lymphoid stromal tissue (asterisk) surrounded and infiltrated by low-grade endometrial carcinoma (arrow)

Final histology identified a focus of well differentiated uterine intramucosal endometrioid adenocarcinoma, without any sign of myometrial invasion (Fig. 1a). Cervix, tubes and ovaries were disease-free. No metastases were found in the peritoneal biopsies or in the pelvic and retroperitoneal removed lymph nodes. However, histology confirmed bilateral inguinal lymph nodes (2 out of 6 overall) positive for endometrial adenocarcinoma (Fig. 1b), with immunohistochemistry positive for CK7, ER, VIM, and negative for CK20 (typical in EC). Moreover, cytology of the peritoneal fluid showed atypical cells.

**Fig. 2:**
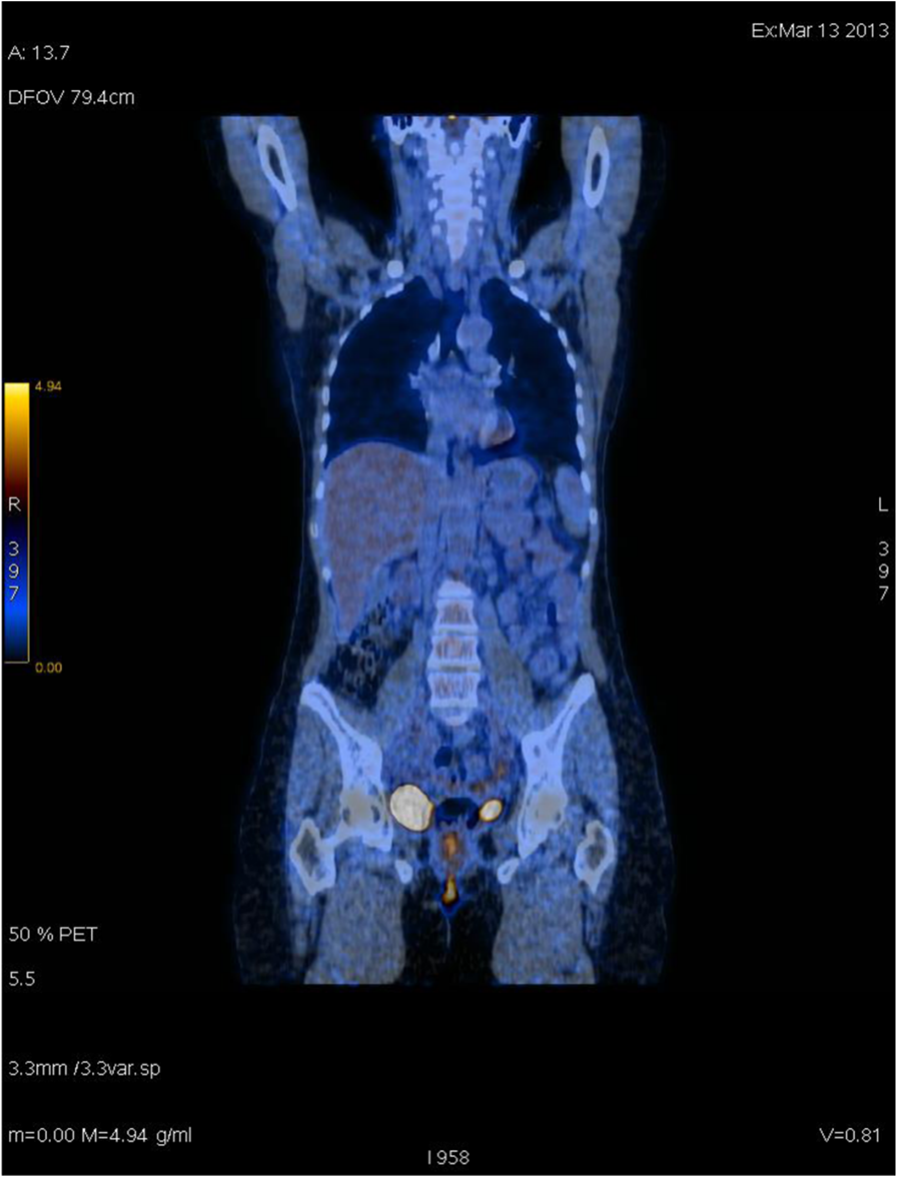
Positron Emission Tomography (PET) showed uptake in the right and left inguinal areas referable to residual disease or inflammation, without other pathological areas

Positron Emission Tomography (PET), performed 5 weeks after surgery, showed uptake in the right inguinal area referable to residual disease or inflammation, without other pathological areas (Fig. 2). The patient was submitted to pelvic external beam radiotherapy (4500 cGy in 25 fractions) with concomitant weekly cisplatin and subsequent boost on the inguinal region (2000 cGy in 10 fractions). Moreover, 4 additional cycles of Cisplatin (70 mg/mq) every 21 days were administered. To date, the patient is disease-free.

In order to confirm histological examination and to understand whether the metastatic inguinal lymph nodes were derived from the EC, we used mitochondrial genome sequencing [6]. Samples of tumor tissue and metastatic lymph nodes were formalin-fixed and paraffin-embedded. Haematoxylin and eosin sections were reviewed to identify paraffin blocks with tumor areas. A control (non tumor) DNA specimen was obtained from the patient’s saliva after collection of informed consent. Whole mtDNA sequencing was performed on EC, metastatic inguinal lymph node and saliva-derived total DNA as previously described [5–7]. We used a PCR-based resequencing system which enables identification of sequence variations in the entire human mitochondrial genome and its control region. Total DNA was used for PCR amplification of 46 overlapping fragments covering the entire mtDNA using a set of 46 primer pairs. Overlapping regions of the mitochondrial genome are amplified with specific primer pairs tailed with universal M13 sequences at their 5′ end to generate resequencing amplicons. Amplicons are then used as templates for quick sequencing using universal M13 primers. All primer pairs are ready to use and anneal at the same temperature. The purified PCR product was used for direct sequencing with BigDye kit version 1.1 (Thermo Fisher Scientific). Sequences were run in an ABI 3730 Genetic Analyzer automated sequencing machine. Electropherograms were analyzed with SeqScape version 2.5 software. Mitochondrial DNA mutations detected in this first phase were confirmed using a second PCR reaction. When the latter showed the same mitochondrial DNA variant of interest, the mutation was confirmed on a second extraction of DNA from the same sample to exclude DNA contamination or sample mix-up. The informative nature of mitochondrial mutations was ascertained by sequencing mitochondrial DNA from saliva.

**Fig. 3:**
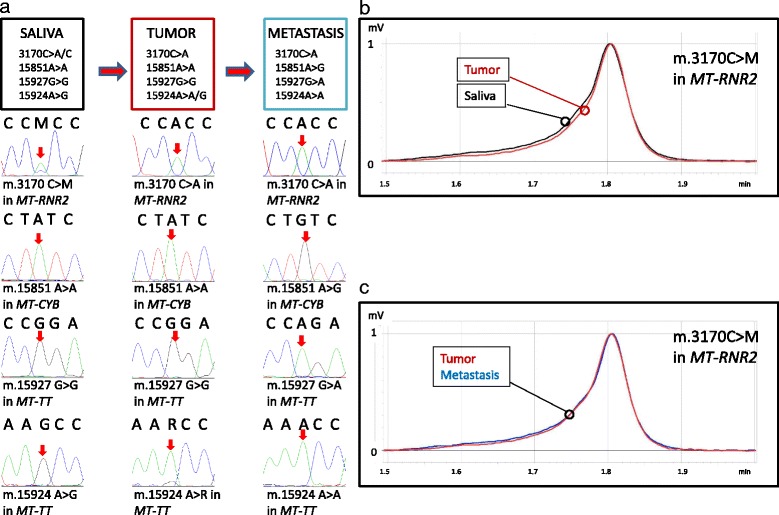
**A-** Sequence analysis of mtDNA variants in saliva, tumor and metastasis. Red arrows indicate the mutated bases.**B-** DHPLC analysis of the m.3170 C > M in MT-RNR2 in tumor and saliva. Homo and heteroduplexes are distinguished based on different retention times. Two elution peaks for saliva (heteroduplex and homoduplex) and a single elution curve for tumor are present. Saliva (black), tumor tissue (red). **C-** DHPLC analysis of the m.3170 C > M in MT-RNR2 in tumor and metastasis. A single elution curve for tumor and metastasis is present. Tumor tissue (red), metastasis (blue)

Sequence analysis was performed with MToolBox [8] and the genomes were deposited in public human mitochondrial database HmtDB [9] with the following identifiers: endometrial tumor: PA_EU_IT_0271, metastasis: PA_EU_IT_0272, saliva: PA_EU_IT_0273. The analysis revealed the presence of several informative variants. The m.3170C > A in the *MT-RNR2* gene was found heteroplasmic in the saliva and homoplasmic both in the tumor and metastatic nodes (Fig. 3a). By using denaturing high pressure liquid chromatography (DHPLC) (Fig. 3b-c), a method shown to be sensitive enough to detect heteroplasmy levels as low as 2% [10] we were able to confirm the heteroplasmy status of these mutations in the saliva. The m.15851A > G in *MT-CYB* and the m.15927G > A in *MT-TT* were found homoplasmic only in the metastasis specimen. The m.15924A > G in *MT-TT* was present in homoplasmy in the saliva, in heteroplasmy in the tumor tissue and was not present in the metastatic lymph nodes, indicating a reverse shift occurring stepwise during progression from primary tumor to nodal metastasis. Similarly, to the latter, the shift to homoplasmy of the m.3170C > A exclusively occurring in the EC and in the metastatic tissues may be considered as a marker of clonal origin of the two tissues.

## Discussion

Usually, EC diffuses by direct extension, transtubal dissemination, lymphatic dissemination, and by hematogenous spread. The risk of adnexal, lymph node, and peritoneal metastasis in patients with well-differentiated EC and no myometrial invasion is extremely low [11, 12]. However, we presented an unusual case of bilateral inguinal LNI from a focal intramucosal well-differentiated endometrioid EC without other sites of metastatic spread. To our knowledge, this is the first case of inguinal lymph node metastases, as first presentation, from intramucous EC, which could be explained in two different ways: 1) lymphatic spread via round ligament of a primitive uterine tumor regressed with hormonal therapy; 2) EC arising from the malignant transformation of ectopic foci of endometriosis. In order to explain the first hypothesis, our patient was submitted to hormonal therapy in two different time frames; progestin for 5 months before accessing our Unit and subsequent GnRH analogue after she refused hysterectomy. Generally, well-differentiated EC expresses hormonal receptors and therefore responds to hormonal therapy such progestins, progesterone, oral contraceptives, selective estrogen receptor modulators, GnRH agonists and aromatase inhibitors. As consequence, these therapies may have led to the control/remission of EC primary site in our patient. Indeed, complete response to hormonal therapies are reported in about 72% of young female patients with early well-differentiated EC who prefer fertility-sparing treatments [13]. In these individuals, the combined use of progestin and GnRH analogues leads to better oncological outcomes. Three different groups have reported four cases of inguinal metastasis as presenting symptom of EC. In 1978, Paulussen et al. [14] described two cases of post-menopausal asymptomatic women with inguinal lymph node metastases, six months and two years before diagnosis of a high grade EC. Scholz et al. [3] reported a case of a 54-year old patient with metastatic mucinous adenocarcinoma in inguinal lymph node as first presentation, associated with a well-differentiated endometrioid adenocarcinoma with the invasion of the inner half of myometrium and positive pelvic and retroperitoneal nodes. In a most recent case report, Shokouh et al. [4] described a premenopausal woman with enlarged right inguinal lymph node. The biopsy showed a metastatic adenocarcinoma of unknown origin. Six months later, a uterine curettage showed a moderately differentiated EC; the patient underwent total abdominal hysterectomy with bilateral salpingo-ophorectomy that revealed a grade 2 adenocarcinoma with invasion of the inner part of the myometrium.

The second hypothesis seems to be less likely. Inguinal endometriosis secondary to the involvement of the extraperitoneal portion of the round ligament is a rare condition, occurring in less than 1% of patients with endometriosis [15]. Moreover, carcinogenesis from endometriosis is a rare event and our patient had no known history of endometriosis and no evidence of ectopic endometrial tissue in any sites.

Due to the peculiarity of this case and for additional diagnostic confirmation of inguinal node metastasis from EC, we performed mtDNA sequencing. MtDNA mutations are extremely common somatic events in human cancers [6], as the mitochondrial genome is more susceptible to mutations occurrence than nuclear DNA. We have previously shown that the detection of a random somatic mtDNA mutation in both EC and metastasis of the same patient may be considered as a marker of clonality of the two lesions, as it is virtually impossible that the same tumor-specific mutation may arise in two independent neoplasms [5]. Similarly, germ-line variants may also be informative when they allow to trace a cell lineage, as it is extremely unlikely that they accumulate to homoplasmy either towards the wild-type or the mutated allele, in independent tumors [16].

**Fig. 4:**
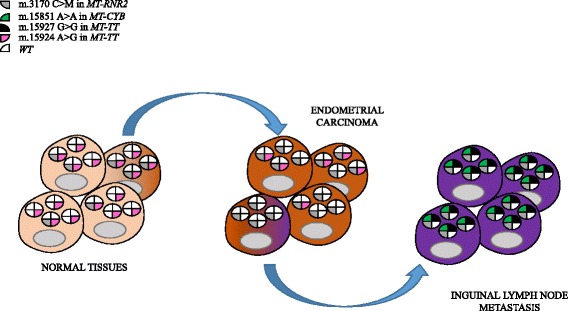
Hypothetical mode of progression from normal cells to metastasis. Each circle within a cell represents wild type mtDNA (white background) carrying different mutations (colored sectors). Cells with grey mutations may be positively selected and pink mutations may start becoming selected against during tumor progression of the primary carcinoma (brown-shaded cell among the normal ones). Subsequently, cells devoid of pink mutations and homoplasmic for the grey mutation may be further selected for their metastatic potential, and they may subsequently accumulate green and black mutations (purple-shaded cells among the carcinoma ones)

Since shift to homoplasmy of the m.3170C > A occurs exclusively in the tumor and in the metastatic tissues, we may assume that the metastatic inguinal lymph node derived from the endometrioid adenocarcinoma. Furthermore, the m.15924A > G was present in homoplasmy in the saliva but the mutation load was lower in the tumor and it was not present in the metastasis, showing a shift to the wild-type nucleotide and suggesting a reversion during tumor progression and metastasis development, likely due to the occurrence of selective pressures. The other two variants, founded exclusively in the metastatic specimen, may be subsequent to the detachment of cancer cells from the primary tumor, hence defining this specific cancer’s lineage (Fig. 4).

## Conclusions

In the literature, several cases of unusual spread of low grade EC have been reported, such as liver, muscle, scalp and cranial bone metastasis. This testifies the unpredictable spread of endometrial neoplasia, beyond current guidelines. In good clinical practice, patient’s history and physical examination should be carefully performed with special attention to inguinal and retro-clavicular lymph nodes regions. Furthermore, in unusual cases, the study of clonality of the lesions with mtDNA sequencing could be performed in order to provide additional informations for a correct diagnosis.

## Abbreviations

CTCAE: Criteria for Adverse Events
DHPLC: Denaturing high pressure liquid chromatography
EC: Endometrial Carcinoma
EIN: Endometrial intraepithelial neoplasia
GnRH: Gonadotropin Releasing Hormon
LNI: Lymph node involvement
MiPEO: Mitochondria in Progression of Endometrial and Ovarian Cancer
mtDNA: Mithocondrial DNA
NSAIDs: Nonsteroideal Anti-Inflammatory Drugs
PET: Positron Emission Tomography
